# Olfactory Preference of *Drosophila suzukii* Shifts between Fruit and Fermentation Cues over the Season: Effects of Physiological Status

**DOI:** 10.3390/insects10070200

**Published:** 2019-07-06

**Authors:** Rik Clymans, Vincent Van Kerckvoorde, Eva Bangels, Wannes Akkermans, Ammar Alhmedi, Patrick De Clercq, Tim Beliën, Dany Bylemans

**Affiliations:** 1 Zoology/Pomology Department, Research Centre for Fruit Cultivation (pcfruit npo), Fruittuinweg 1, B-3800 Sint-Truiden, Belgium; 2 Department of Plants and Crops, Faculty of Bioscience Engineering, Ghent University, Coupure Links 653, B-9000 Ghent, Belgium; 3 Department of Biosystems, KU Leuven, Decroylaan 42, B-3001 Heverlee, Belgium

**Keywords:** *Drosophila suzukii*, seasonal changes, monitoring, apple cider vinegar, fruit volatiles, seasonal morphology, mass trapping, olfactory preference, nutritional state, reproduction

## Abstract

Worldwide monitoring programs of the invasive fruit pest *Drosophila suzukii* Matsumura (Diptera: Drosophilidae), using fermentation baits like apple cider vinegar (ACV), revealed a counterintuitive period of low trap catches during summer, followed by an autumn peak. In this study, we demonstrate that ACV baited traps indeed provide a distorted image of the *D. suzukii* population dynamics as it is possible to capture higher numbers during this “low capture period” with synthetic lures. It was hypothesised that the preference of *D. suzukii* populations for fermentation cues like ACV is most pronounced during autumn, winter and spring, while the flies prefer fresh fruit cues during summer and that this seasonal preference is related to the changing physiology of the flies over the season. To test this hypothesis, the preference between fermentation cues (ACV) and host fruits (strawberries) and the effect of physiology (sex, seasonal morphology and feeding, mating and reproductive status) was investigated both in olfactometer laboratory experiments and a year-round field preference experiment. In olfactometer experiments we demonstrated that protein deprived females, virgin females with a full complement of unfertilised eggs and males show a strong preference for fermentation cues while fully fed reproductive summer morph females generally prefer fruit cues. These findings indicate that *D. suzukii* is attracted to fermentation volatiles in search of (protein-rich) food and to fruit volatiles in search of oviposition substrates. Winter morph and starved females displayed indiscriminating olfactory behaviour. In the field preference experiment, the hypothesised seasonal shift between fermentation and fruit cues was confirmed. This shift appeared to be highly temperature-related and was similarly observed for summer and winter morphs.

## 1. Introduction

Spotted wing Drosophila, *Drosophila suzukii* Matsumura (Diptera: Drosophilidae), native to Southeast Asia [1,2,3], recently invaded major fruit production regions across Europe, as well as North and South America [3,4,5,6,7,8,9,10]. Its widespread establishment was favoured by a broad climatic tolerance [6,11,12,13,14,15], the absence of efficient native natural enemies [16,17,18] and its large host range including non-crop hosts as well as high-value crops (stone fruits, blueberry, strawberry, currants, raspberry, blackberry, plums, grapes and apricots) [19,20,21,22,23,24,25]. This makes *D. suzukii* a serious economically damaging fruit pest [8,9,26] and its invasion has strongly disrupted integrated pest management (IPM) programs, as growers are forced to adopt calendar spraying with (broad-spectrum) insecticides [7,27,28], due to a lack of reliable monitoring tools that accurately predict fruit infestation [29,30,31].

Monitoring programs using apple cider vinegar (ACV) based baits result in a rather unusual population dynamics profile. Throughout spring and early summer only limited numbers of *D. suzukii* adults are trapped [5,32,33,34,35,36,37]. This has even been referred to as the “low capture period” (LCP) [32]. Trap counts only start increasing in late summer, to reach peak levels in autumn [5,32,33,34,35,36,37,38]. This pattern has been observed in several European countries, including Italy [32,36,37,38], Switzerland [33], Germany [35], Spain [34] and Belgium [5]. A similar trap catch pattern was observed in the United States, in regions with a temperate climate (Michigan, Washington and Oregon) [6,39]. In the USA, a similar LCP was also detected using other fermentation or wine based baits, but with trap catches slightly advanced in the year [40,41]. In climatologically warmer states (North Carolina and especially California) an earlier or different capture pattern was observed [1,6,39]. 

A number of reasons have been suggested for this unusual population profile: Several researchers in warmer climates (e.g., California) attribute this LCP to excessive heat during summer [6,39,42,43]. Although this reason may be valid in warmer regions, it cannot be the only explanation, as the same phenomenon was clearly observed in temperate climates [5,32,33,34,35,36,37,38]. It also has been stated that trap counts do not represent the actual population density, since they are influenced heavily by weather (cold, heat, rain) and the surroundings of the trap (presence of fruits, hibernation habitats) [35,36,39,43,44]. Weather can influence both activity as well as survival (and thus population density) of the fly. Because bait trapping is a sampling method with an activity-density bias, trap counts should be interpreted with caution [45,46].

The direct competition between ACV baited traps and fruits seems to be of particular importance for the LCP. Numerous authors describe the poor correlation between fruit infestation and trap catches [1,29,31,35,47]. The flies seem to prefer the ripe fruits markedly more than the ACV mixture, resulting in an under-representation of fly density during the fruiting season in ACV baited traps [35]. Similarly, after the fruit harvest, captures sharply increase, due to newly emerging adults and the end of the fruit-lure competition [48]. Although fruit presence can influence ACV trapping efficiency, this does not seem to be the single cause of the LCP, since there are examples of rising trap catches in the presence of fruit at the end of summer [33,37,38,43].

The present study focuses on an additional factor influencing the population profile obtained with ACV: The possibility that *D. suzukii* preference is dependent on the physiological state of the fly at any particular time. The influence of fly physiology on food preferences and olfactory responses is well documented in *Drosophila melanogaster* [49,50,51,52,53,54,55], but understudied in *D. suzukii* up to date. However, clear effects have been observed of seasonal morphology [56], recent (4 to 5 h) mating [57], reproduction and nutritional status [58] on the responsiveness to odours in no-choice experiments (either using traps [56,58] or wind tunnels [57]). Wong et al. [58] complemented their no-choice cage or greenhouse trap assays (i.e., using a single fermentation based bait/attractant) with a multichoice cage trap assay investigating the relationship between oogenic status (number of mature eggs) and the preference for either a fermenting bait, raspberry essence or water. In the present study, true choice experiments were carried out, using actual fruits and ACV as a standard bait, with the aim to specifically investigate whether olfactory preference of *D. suzukii* for ACV versus ripe fruit is altered by physiological state. This relevant dual choice (i.e., strawberry fruits vs. ACV) as affected by the fly’s physiological state was studied both in the laboratory and in a yearlong field experiment allowing to detect seasonal shifts in preference that could be linked to the described LCP in ACV-monitoring programs.

## 2. Materials and Methods

### 2.1. Field Comparisons of ACV Baits with Synthetic Lures

#### 2.1.1. Experimental Sites

Four field experiments were conducted in two subsequent years. In 2015, two experiments were performed at commercial farms, one in *Vaccinium corymbosum* (blueberry cv. Dixie and Blue Crop, surrounded by wild habitats for *D. suzukii* including wild blackberry; Koersel, Belgium, 51°4.117′ N, 5°16.427′ E) and another in *Rubus ursinus* (commercial, rain shelter protected blackberry cv. Loch Ness; Spalbeek, Belgium, 50°55.912′ N, 5°12.857′ E). In 2016, another two experiments were done in sweet cherry, *Prunus avium*. The first experiment was executed in two abandoned cherry orchards (2 replicates in sweet cherry cv. Kordia, Regina, Karina and Samba; Bevingen, Belgium, 50°48.070′ N, 5°11.004′ E and 2 replicates in sweet cherry cv. Kordia, Regina, Schneiders Späte Knorpelkirsche and Lapins; Metsteren, Belgium, 50°50.651′ N, 5°10.661′ E) and the second in a commercial cherry orchard (sweet cherry, cv. mainly Kordia, Regina and pollinators; Metsteren, Belgium, 50°50.185′ N, 5°10.645′ E).

#### 2.1.2. Experimental Design

In each experiment, 8 red traps with a transparent lid coated on the interior with 15 mg of deltamethrin (Decis™ Trap Suzukii, experimental prototype, Bayer Crop Science, Monheim, Germany) were deployed on 17 and 28 July, and 15 and 18 April, in blueberry, blackberry, commercial and abandoned cherry, respectively. The traps were filled with either 200ml of apple cider vinegar (ACV) based bait; in 2015 this was pure ACV (cider vinegar 5% acidity, Burg, Vinaigrerie Fuchs, La Tremblade, France), in 2016 this was a mixture of 74% w/w ACV (cider vinegar 5% acidity, Burg, Vinaigrerie Fuchs, La Tremblade, France), 24% w/w red wine (Bag-in-box, Aldi, Erpe-Mere, Belgium) and 2% w/w dark brown candi sugar, Tiense Suikerraffinaderij (Candico), Merksem, Belgium) or an experimental lure (Synthetic Decis™ Trap Suzukii lures DS D TM 31.15 (2015) and DS D TM 55.16 (2016), Bayer Crop Science, Monheim, Germany). All experimental sites were divided into 4 blocks (replicates), each having one trap of each attractant with a minimal distance of 15m between the traps (within a block and between blocks). Weekly, all traps were shifted in position after emptying to avoid location-based bias (it was ensured that each attractant occurred equally at all monitoring positions). 

#### 2.1.3. Assessments

Traps were emptied every 6 to 10 days in the blueberry, 6 to 8 days in the blackberry and 7 days in the cherry experiments. Identification and counting of *D. suzukii* males and females was done using a binocular microscope.

### 2.2. Olfactometer Experiments with ACV versus Strawberries: The Effect of Physiology

#### 2.2.1. Insects

##### Stock Culture

The *D. suzukii* culture used in the laboratory experiments originated from multiple collections of adults in a private garden (Gentbrugge, Belgium, 51°1.522′ N, 3°46.093′ E), during March and April 2018. The laboratory colony was maintained, for a maximum of 12 months prior to the experiments, in polystyrene Drosophila vials (Greiner Bio-One™ Insect Breeding Conical Container, 217101) on a cornmeal-yeast-agar diet (42 g/L fresh yeast, *Saccharomyces cerevisiae*, Algist Bruggeman; 55 g/L white table sugar, Suikerraffinaderij Tienen; 90 g/L crushed cornmeal, Aveve; 2 g/L Ethyl 4-hydroxybenzoate 99%, Alfa Aesar; 9 g/L agar powder, VWR chemicals and 910 g/L tap water). The vials were stoppered using foam stoppers (Greiner Bio-One™ Ceaprenstop, diam. 36 mm, 330070) and kept in a plant-growth chamber at 22 ± 1 °C, 60 ± 11% RH, and a 16:8 L:D photoperiod. Cohorts with different physiological characteristics were generated to test the effect of seasonal morphology and of feeding, mating, and reproductive status on preferences in olfactometer experiments.

##### Feeding Status

Starved, sugar-fed and artificial medium-fed flies were compared: Upon emergence, flies were maintained on a cornmeal-yeast-agar diet for 2 to 6 days. In the last 24 h prior to the experiment, the flies for the ‘starved’ treatment were transferred to polystyrene Drosophila vials with cotton wool soaked with tap water. For the “sugar-fed” treatment, the cotton wool was soaked with 10% sugar solution. The “medium-fed” treatment was established by transferring flies to vials with the cornmeal-yeast-agar diet. At the start of the experiment, the flies were between 3 and 7 days old. Each olfactometer contained 10 female and 10 male flies. Four olfactometers (replicates) per treatment were simultaneously used per experiment and the whole experiment was repeated 3 times in a row (n = 12).

##### Mating Status

Mated and virgin female flies were compared: Flies were collected within 3 h after emergence (sexually immature) and immediately sexed using a stereo microscope without anesthetizing. Half of the females were separated from the males to prevent mating. The other half were kept with an equal number of males to allow ad libitum mating. All flies were maintained in polystyrene Drosophila vials on a cornmeal-yeast-agar diet for 7 days prior to the test. Each olfactometer contained 10 female flies. Six olfactometers (replicates) per treatment were simultaneously used per experiment and the whole experiment was repeated 3 times in a row (n = 18).

##### Reproductive Status

Protein deprived females (sugar-fed, i.e., not merely deprived of protein but for simplicity and due to higher relevance of protein [59,60] further referred to as such), with a 24 h protein repletion period and protein fed females were compared. This was done to compare females of different reproductive stages: Sugar-fed females were assumed to show no or limited egg development [58,61], medium-fed females were assumed to show plenty of mature eggs in the ovaries and sugar-fed flies allowed 24 h access to artificial medium (to reduce nutrient balancing behaviour) preceding the experiment were assumed to still have limited egg development while showing less nutrient balancing behaviour. To confirm these assumptions, all females were dissected after the experiment to count the vitellogenic oocytes. Males and females were collected within 3 h after emergence and maintained in polystyrene Drosophila vials with either standard cornmeal-yeast-agar medium or cotton wool soaked with a 10% sugar solution for 6 days. All flies were transferred to new Drosophila vials 24 h prior to the test: Half of the sugar-fed flies were transferred to cornmeal-yeast-agar medium (i.e., 24 h protein repletion), the other half were provided again with a 10% sugar solution (i.e., protein deprived). The flies kept on artificial medium were transferred to new vials with fresh artificial medium (i.e., protein fed). At the start of the experiment the flies were 7 days old. Each olfactometer contained 10 female flies. Four olfactometers (replicates) per treatment were simultaneously used per experimental run and the whole experiment was repeated 3 times in a row (n = 12).

##### Seasonal Morphology

Winter and summer morphs were compared. Both morphs were reared in polystyrene Drosophila vials on a standard cornmeal-yeast-agar diet. Summer morphs were maintained at 22 ± 1 °C, 60 ± 11% RH and a 16:8 L:D photoperiod. For winter morph rearing, adult females of summer morphs were first allowed to oviposit for 24 h and were then removed. Next, the vials were transferred to winter morph generating conditions (9.4 ± 0.2 °C, 72 ± 11% RH, 12:12 L:D) [62]. Approximately 2 months later winter morph adults started emerging over a period of about two weeks. Hence, these tests were executed with flies of unsynchronised age (it is worth noting that summer and winter morphs are also not readily comparable in age due to the discrepancy in longevity): Both summer and winter morphs were randomly selected from a mixed age population. Seven days prior to the test, adults of both morphs were transferred to new vials with cornmeal-yeast-agar medium and maintained at 22 ± 1 °C, 60 ± 11% RH and a 16:8 L:D photoperiod to allow the winter morphs to acclimate, break quiescence and acquire a similar stage of egg development as the summer morphs [27,59,63,64,65]. To confirm the latter, all females were dissected after the experiment to count the vitellogenic oocytes. Each olfactometer contained 10 female and 10 male flies. Six olfactometers (replicates) per treatment were simultaneously used per experiment and the whole experiment was repeated 3 times in a row (n = 18).

#### 2.2.2. Experimental Set-up

##### Olfactometer

**Figure 1:**
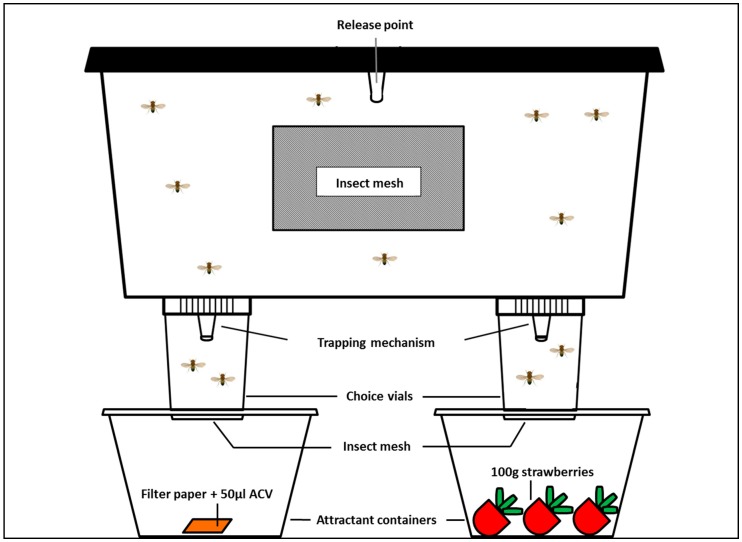
Side-view of the four-arm olfactometer. A fixed number of *D. suzukii* flies are released in the middle of the box. The trapping mechanism prevents escape once a fly has chosen. Contact with the attractant is prevented by insect mesh between the choice vials and the attractant containers.

The main part of the four-arm olfactometers used in the experiments consisted of a customised transparent polypropylene storage box (Starplast storing box, 30 × 15 × 17 cm, with lid, Action Belgium BVBA). Five circular ventilation holes (4 cm ø, four holes in the lid and one in the bottom) and one rectangular hole per side (5 × 8 cm) were cut out and covered with insect mesh (UV stabilised polyethylene insect net, mesh size 0.4 × 0.4 mm). The mesh was fixed using contact glue (Universal contact glue, Bison International), and allowed to air-dry long before the experiments. A circular opening (11 mm ø) was heat punched in the center of the lid using a heated metal cylinder. This served as an entry for release of the flies and was closed off using a centrifugal tube (1.5 mL, PP, graduated, attached cap, natural, 616201, Greiner Bio-One BVBA, Vilvoorde, Belgium. A hole (4 cm ø) was created using a heated iron cylinder (heat punched) through the bottom of four polypropylene vials (urine collection container with screw cap, 100 mL, 75 × 65 mm, 216-0288, VWR International BVBA, Heverlee, Belgium). These vials served to collect flies with different “choices” in the olfactometer. The bottom of these vials was pushed into a round hole (4.6 cm ø), that was heat punched in the lid of a round plastic container (Microwavable container, PP, 500 mL, 12 cm ø, with white lid T20RD, Werti Packaging, Sint-Truiden, Belgium), containing the tested attractants. A piece of insect mesh was fixed in between both containers. This setup allowed the flies to make choices without contact with (or oviposition on) or visual stimulation by the attractant. Next, the screw caps of the vials were glued 4 cm inwards from each corner. A circular opening (11 mm ø) was heat punched through the screw cap and bottom. After cooling, a modified microcentrifuge tube (1.5 mL, PP, graduated, attached cap, natural, 616201, Greiner Bio-One, BVBA, Vilvoorde, Belgium) was pushed in the circular opening. Prior to installation the bottom and cap were cut off, creating a small funnel with a diameter of 5 mm. These tubes served as the trapping mechanism. The olfactometer design is visualised in Figure 1.

##### Procedure

Two “attractant containers” in the bottom of the olfactometer were loaded with 50 µL apple cider vinegar (cider vinegar 5% acidity, Lot E0417012, Burg, Vinaigrerie Fuchs, La Tremblade, France) on a 4 cm^2^ square piece of filter paper (Whatman^®^ quantitative filter papers, ashless, Grade 589/1 black ribbon, GE Healthcare Life Sciences Europe GmbH, Diegem, Belgium, circles, diameter 150 mm, pack of 100) and two with 100 ± 5 g of strawberries (*Fragaria x ananassa*, cv. Sonata, all originated from one single greenhouse, 50°49.544′ N, 5°34.760′ E). The strawberries were small sized (9.5 ± 1.7 g, n = 30). Per experiment, twelve olfactometers were placed in a ventilated plant growth chamber at 22 ± 0.1 °C, 75 ± 0.8% RH and a 24:0 L:D photoperiod. The experiments were started by collecting ten female and, where relevant, ten male *D. suzukii* adults (using an insect aspirator) and releasing them in the center of the olfactometer. Flies of different treatments were tested simultaneously and over the experiments, treatments were placed equally on each position in the growth chamber. After three, six and 24 h, the choices were assessed by counting the number of female (and male) *D. suzukii* per choice trap. After 24 h, the flies were collected and the attractant containers were disposed of and replaced. Subsequently, all flies were stored in saline solution (0.9% w/v NaCl) with one drop of detergent (Afwasmiddel PUUR 0% perfume, colouring and parabens, Albert Heijn) per liter. Samples were kept in a refrigerator (maximum 7 °C) for the maximum of two weeks until dissection. Each female was placed separately in a small drop of the saline solution in the shallow well of the lid of a 24–well plate, placed on a black background to facilitate dissection. The thorax was held with precision forceps while the abdomen was opened with a hooked micro dissecting needle. With two micro dissecting needles, the ovaries and uterus were examined. Ovarioles were separated to determine the stages of oogenesis, making a distinction between females with undiscernible and strictly previtellogenic ovarioles and females with vitellogenic oocytes in the ovaries. Vitellogenic oocytes and mature eggs (both in ovaries and uterus) were counted.

### 2.3. Year-Round Field Olfactory Preference Experiment with ACV versus Strawberries

#### 2.3.1. Experimental Site

This field experiment was performed in an abandoned cherry orchard in 2017. The orchard in Metsteren (Sint-Truiden, Belgium, 50°50.655′ N, 5°10.667′ E, 0.35 ha) was bordered by favourable *D. suzukii* overwintering sites, including woodlands and urban refuges. The field preference experiment ran from October 2016 to December 2017.

#### 2.3.2. Experimental Design

Twenty red insect traps (Droso-trap^®^, Biobest Group NV, Westerlo, Belgium) were deployed in the orchard. The interior of the transparent lid was coated with deltamethrin (15 mg/lid), allowing trapping without a drowning solution. Ten traps were filled with ACV and ten with strawberries. The ACV (cider vinegar 5% acidity, Lot E0417012, Vinaigrerie Fuchs, La Tremblade, France) was placed in polystyrene Drosophila vials (Greiner Bio-One™ Insect Breeding Conical Container, 217101). The vials were completely filled and stoppered using foam stoppers (Greiner Bio-One™ Ceaprenstop, diameter 36 mm, 330070) allowing continuous contact of the vinegar with the stopper. Hence only the insecticide coated lid could generate mortality of *D. suzukii* and differences in mortality due to drowning in the apple cider vinegar were eliminated. The ripe and fully red coloured strawberries (50 g, Fragaria x ananassa, cv. Cléry, Sonata, Malling Centenary, Elsanta, Verity and strawberries of unknown cultivar purchased on the local market, subject to availability) were placed in a plastic deli container (250 mL, rectangular, polypropylene, with lid, Werti packaging, Sint-Truiden, Belgium). A hole (6 × 4 cm) was cut in the lid and screened with insect mesh (UV stabilised polyethylene insect net, mesh size 0.4 × 0.4 mm). The traps were emptied and the strawberries and vinegar were replaced with a mean interval of 7.7 ± 1.8 days from October 2016 to October 2017, but in November and December 2017 longer intervals were adopted (17.8 ± 8.7 days). The trapping intervals and strawberry varieties were incorporated in the statistical analysis.

#### 2.3.3. Assessments

The number of trapped *D. suzukii* flies were counted and differentiated based on sex and seasonal morphology, using the characteristics described by Shearer et al. [62]. The differentiation between summer and winter morphs was based on a visual rating of the melanisation of the third abdominal segment for males and the fourth for females. For the winter morph, the melanisation of these segments is complete, but in summer morphs at maximum only half of the segment is melanised. Weather data were obtained from a Mety (Bodata, Dordrecht, Netherlands) weather station within a 9 km range of the experimental site. Values for daylength were obtained from the Royal Observatory of Belgium [66].

### 2.4. Data Analysis

For the analysis of the field comparison of synthetic lures and ACV based baits, a generalized mixed model with Poisson distributed errors and a log-link function is used to model the fly counts in each trap. The row number was included as random intercept to account for the expected influx gradient of a neighbouring semi-natural environment. As fixed effects the sampling date, attractant type and sex are introduced in the model. Both the attractant type and sex are assumed to be nested in the sampling date, making comparisons within each sampling date possible.

In the laboratory experiments, for each olfactometer (12 or 18 per treatment, divided over 3 runs) at each assessment time (3, 6 and 24 h) a preference index (PI) was calculated with PI = (number of flies in the 2 strawberry loaded arms − number of flies in the 2 ACV loaded arms)/(number of flies in the 2 strawberry loaded arms + number of flies in the 2 ACV loaded arms) [67]. A PI calculated from one olfactometer containing 10 flies of the same sex was considered as one observation (n = 1) for further analysis. A linear mixed model was used to analyse the calculated PI’s. Both replication and location (in the growth chamber) are introduced as random intercepts. As fixed effect the observation time and the treatment are included in the model. The treatment is assumed to be nested in the observation time to allow a per-time comparison. 

A mixed logistic regression model with logit-link function was adopted to model the effect of oogenesis on the preference for strawberry, regarded as a “success” against ACV. Both replication and location are introduced as random intercepts. Fixed effects included in the model are the number of mature eggs or the number of vitellogenic oocytes and the vertical position in the climate chamber (upper or lower shelf). 

For the field preference experiment, a mixed logistic regression model with logit-link function was selected to model the preference for strawberry (“success”) against ACV. As a random intercept, the strawberry cultivar was introduced. The fixed effects in the model are trapping interval, seasonal morphology, sex of the fly, average temperature and average relative humidity in the given trapping interval and daylength. To account for possible population size effects, the latter three are assumed to be nested in the seasonal morphology.

Unless stated otherwise, data provided in text are always the sample mean ± the sample standard deviation. 

## 3. Results

### 3.1. Field Comparisons of ACV Baits with Synthetic Lures

**Figure 2:**
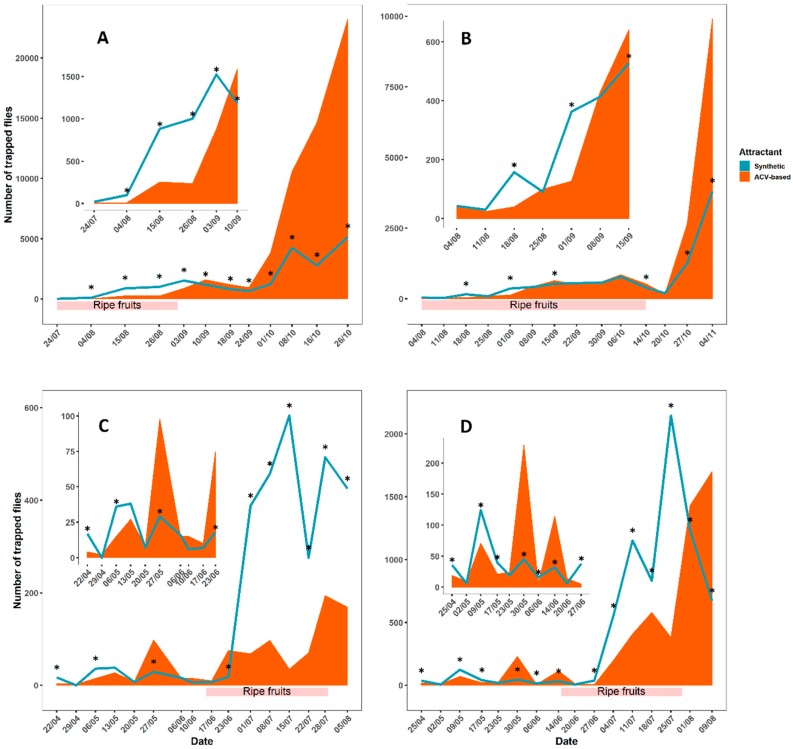
Field observations with synthetic lures and ACV baited traps during spring and summer in (**A**) Blueberry in 2015, (**B**) Blackberry in 2015, (**C**) Cherry (commercial) in 2016, and (**D**) Cherry (abandoned) in 2016. Each date on the horizontal axis is a sampling date for which the line graphs depict the number of *D. suzukii* adults captured in four traps over the interval between that and the previous sampling date. Asterisks indicate significant differences (*p* < 0.05) between the estimated marginal means of the two attractants. For each graph, a zoomed section of the first part of the experiment is given, the presence of ripe fruits on the crop is represented by the bar under the plot.

In all four field comparisons, the synthetic lure demonstrated that the typical low captures during summer with ACV loaded traps are an underestimation of *D. suzukii* abundance (Figure 2). The synthetic lure was not only consistently earlier in the detection of the flies, it reached 4 to 16 times higher *D. suzukii* trap catches during July and August. In blueberry (Figure 2A), a substantial (i.e., mean number per trap per week >5) first detection of *D. suzukii* with the synthetic lure was noted 2 weeks prior to ACV. The synthetic lure significantly outperformed ACV from the 4th of August till the 3rd of September, with ninefold and fourfold trap catches on the sampling of the 4th and 26th of August, respectively. From September onwards, ACV significantly became the best performing attractant until the end of the experiment (late October). In September, ACV consistently caught about 1.4 times more *D. suzukii* than the synthetic lure. This difference became greater in October, with five- and fourfold trap catches on the last two sampling dates. 

In blackberry (Figure 2B), a similar shift in performance could be noted from the synthetic lure in summer towards ACV in autumn. During the cropping season, the synthetic lure reached significantly higher trap catches (fourfold on the 18th of August and threefold on the 1st of September). The synthetic lure and ACV start to coincide in terms of catches from early September until early October, with the exception of a significantly higher catch by ACV on the 15th of September. Similar to the experiment in blueberry, a marked shift in performance towards ACV was observed in October (14th) and November (4th), with significantly higher trap catches (fivefold and threefold, respectively). 

The experiments in cherry were initiated at the end of April (about 50 days before fruit ripening) and followed through until the beginning of August (i.e., the end of cropping season). In the commercial cherry orchard (Figure 2C) traps with the synthetic lure had a substantial first detection of *D. suzukii* 3 weeks earlier than traps with the ACV based bait. On these early sampling dates (22th of April and 6th of May) the synthetic lure had significantly higher catches than ACV. During the remainder of spring, the synthetic lure and ACV did not differ in terms of catches except for a significantly higher number of flies in ACV on the 27th of May and the 23th of June. Only in early July (when general trap counts increase) a very clear difference in trap catches in favour of the synthetic lure was noted again. From the 1st of July till the end of the experiment (5 August), the synthetic lure significantly outperformed ACV (with a maximum of a sixteenfold trap catch on the 15th of July). 

In an identical experiment in the same year in abandoned cherry orchards, very similar trends were observed (Figure 2D): A one week earlier substantial first detection of *D. suzukii* and a coinciding spring capture pattern with the exception of some alternating significant differences. Likewise, a period of higher catches in synthetic lure traps commenced at the start of July (4th). This period wherein the synthetic lure significantly outperformed ACV lasted until the end of July (with a maximum of a fourfold ACV trap catch on the 25th of July). In contrast with the commercial cherry orchard experiment, the catches with the synthetic lure here decreased while catches with ACV increased during the remainder of the experiment (until the 9th of August), with ACV being the better attractant on the last two sampling dates.

### 3.2. Olfactometer Experiments with ACV versus Strawberries: The Effect of Physiology

#### 3.2.1. Feeding Status

**Figure 3:**
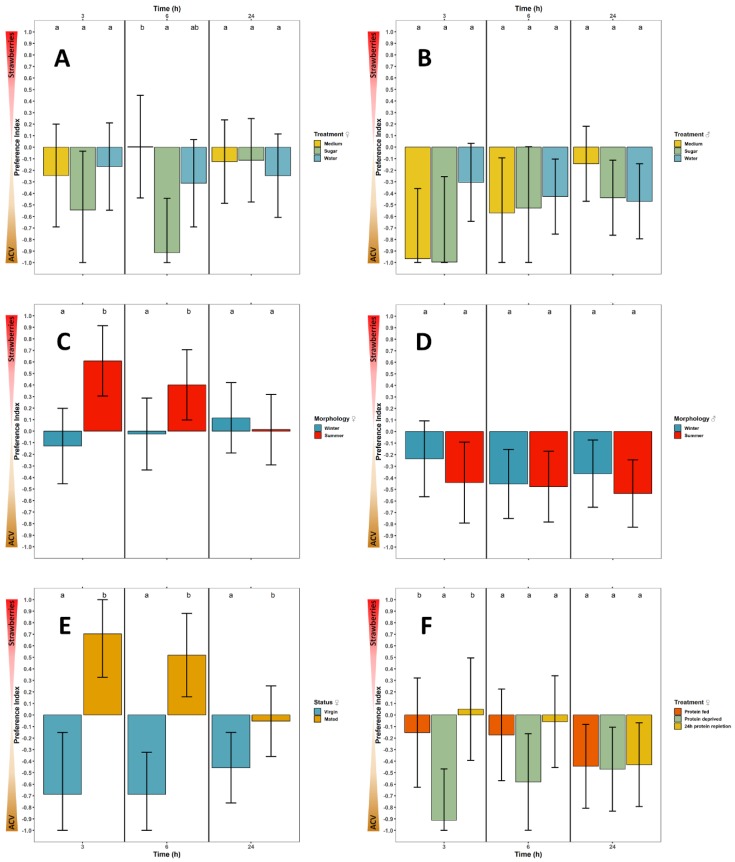
The effect of physiology on the preference of *D. suzukii* between apple cider vinegar (ACV) and strawberries is investigated in olfactometer experiments focused on: (**A**) Females of different feeding status, (**B**) males of different feeding status, (**C**) females of different seasonal morphology, (**D**) males of different seasonal morphology, (**E**) females of different mating status and (**F**) females of different reproductive status, acquired with different diet treatments. Bar graphs show the estimated marginal mean and confidence interval of the preference index. When exceeding the theoretical interval [−1,1], the confidence interval is cropped to its maximum (minimum) level. Different letters denote significant differences between treatments.

At 3 h after the introduction of flies in the olfactometer, all female flies had a slight preference for ACV although no significant differences were observed among treatments (Figure 3A). After 6 h, female medium-fed *D. suzukii* displayed no preference, whereas sugar-fed and starved females (access to water only) strongly (estimated PI ~ −0.9) and moderately (estimated PI ~ −0.3) preferred ACV, respectively. At 6 h, the preference for strawberries of the medium-fed flies was significantly higher than that of the sugar-fed flies. At 3 h and 24 h no significant differences were noted between females of different feeding status, all preferring ACV moderately (estimated PI > −0.25) with the exception of sugar-fed flies which already showed a clear preference (estimated PI about −0.5) for ACV at 3 h. Starved flies were clearly more responsive early in the experiment (Table S1). The sample means of vitellogenic oocytes counted in dissected females after the experiment were 15.8 ± 9.1 for the medium treatment (n = 121), 10.8 ± 9.9 for the sugar treatment (n = 113) and 9.2 ± 9.5 for the water treatment (n = 122). The number of vitellogenic oocytes was significantly higher in medium-fed flies than in starved and sugar-fed flies whereas there was no difference between the latter two. 

Male *D. suzukii* all showed a preference for ACV, regardless of treatment and time of assessment and no significant differences could be observed between treatments (Figure 3B). At 3h, medium and sugar-fed males showed a strong preference (estimated PI < −0.9) for ACV, while starved males only moderately preferred ACV (estimated PI about −0.3). At 24 h, the preference for ACV was low (estimated PI > −0.2) in medium-fed flies and still clear in sugar-fed and starved males (estimated PI ~ –0.4). Here again, starved flies were clearly more responsive early in the experiment (Table S1).

#### 3.2.2. Seasonal Morphology

After 3 and 6 h, female winter and summer morphs displayed significant differences in preference: Female winter morphs slightly preferred ACV (estimated PI > −0.15), while summer morph females clearly preferred the strawberries (estimated PI > 0.4) (Figure 3C). At 6 h, both treatments already start converging and at 24 h, the difference has disappeared, with both morphs hardly showing preference. The sample means of vitellogenic oocytes counted in females after the experiment were 19.7 ± 12.6 for the winter morphs (n = 179) and 22.3 ± 11.1 for the summer morphs (n = 173). The difference in number of vitellogenic oocytes between morphs was small but significant. No significant differences were observed between male winter and summer morphs as both showed a preference for ACV at all assessment times (Figure 3D). Winter morph males had a tendency of being slightly less attracted by ACV than summer morph males. The responsiveness of both morphs at each assessment is given in Table S1.

#### 3.2.3. Mating Status

At all assessment times (3, 6 and 24 h), a significant difference in preference between virgin and mated females occurred (Figure 3E). The virgin flies always had a strong preference for ACV (estimated PI < −0.4), while mated flies showed a clear preference for strawberries at 3 and 6 h (estimated PI > 0.5). At 24 h, mated flies slightly preferred ACV (PI > −0.1), but their preference still significantly differed from the more pronounced ACV preference of the virgin females. The responsiveness of both the virgin and mated females at each assessment is given in Table S1. The sample means of vitellogenic oocytes counted in females after the experiment were 36.6 ± 16.1 for the virgin females (n = 178) and 33.2 ± 10.2 for the mated females (n = 178). The difference in number of vitellogenic oocytes between females of both statuses was small but significant. Virgin flies showed egg retention as no oviposition was noted during their life and no eggs were found in the uterus. 

#### 3.2.4. Reproductive Status

At 3 h, protein deprived female *D. suzukii* had a strong preference for ACV (estimated PI about −0.9) that significantly differed from that of protein fed and protein replenished females (Figure 3F). The latter two had no pronounced preference and did not significantly differ from each other. At later assessment times no significant differences between treatments were noted although at 6h the same trends as at 3h could still be observed, while at 24 h, females of all treatments showed a very similar preference for ACV. The responsiveness of all treatment groups at each assessment is given in Table S1. The sample means of vitellogenic oocytes counted in females after the experiment were 29.1 ± 9.9 for the protein fed females (n = 112), 2.2 ± 3.6 for the protein deprived females (n = 120) and 5.7 ± 3.6 for the protein repletion females (n = 118). The differences between the three treatments were significant (Figure S1) as expected. 

#### 3.2.5. Oogenic Status

Analysis of the data gathered for all females over all experiments, based on the status of oogenesis and the choice of each female after 24 h, showed no relation between the number of vitellogenic oocytes or mature eggs and the preference for strawberry. 

### 3.3. Year-Round Field Olfactory Preference Experiment with ACV versus Strawberries

Winter morph *D. suzukii* were the predominant flies during the whole year, with peaks in spring (mostly females, only 6% males), summer and autumn. The trap catches in late autumn, winter and spring were almost solely the result of ACV-baited traps, whereas the high summer catches almost exclusively originated from strawberry baited traps. In early autumn the trap catches were still mostly from the strawberry traps but with a notable proportion from the ACV-baited traps. Summer morph *D. suzukii* were trapped in high numbers from early summer till early autumn, mainly in strawberry baited traps. During late autumn, a lower number of summer morphs were still trapped but this time almost exclusively with ACV. 

**Figure 4:**
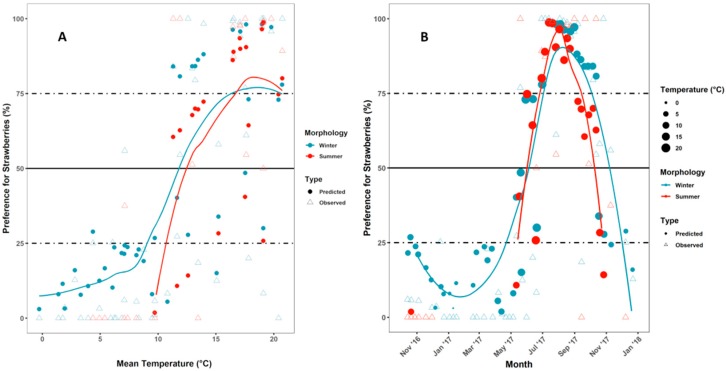
A field preference experiment was done from the 14th of October 2016 till the 22nd of December 2017. Dots are the estimated values from the model, triangles the actual observations. The line is a local regression line (LOESS) and does not represent the underlying logistic function. (**A**) Preference for strawberry (over ACV) in relation with the mean temperature during the trapping interval and (**B**) preference for strawberry (over ACV) over the year. For B, the size of the dot relates to the mean temperature during the interval preceding the corresponding sampling date.

Based on odds ratios, the variables “sex” and “trapping interval” had limited influence on the preference of *D. suzukii*. The effect of the seasonal morphology of the flies was much more pronounced. For both morphs, the mean temperature has the highest odds ratio, indicating that an elevated temperature will increase the probability of a fly to choose strawberry over ACV. Figure 4A shows for both morphs a clear relation between the mean temperature and the modelled preference rate for strawberry. Winter morph flies trapped at low mean temperatures showed a strong preference for ACV, the sigmoid shape of the local regression line suggesting an infliction point around 12 °C. Summer morph flies followed the same tendency, but for this morph no estimated values could be calculated at the lowest temperatures due to the limited occurrence of summer morphs in this climatic range (i.e., only some in autumn and early winter). Between the mean temperatures of 15 and 20 °C a maximum preference rate for strawberry was noted for both morphs. Other, be it less pronounced, related variables were relative humidity and daylength (Figures S1 and S2, respectively). However, given the observational nature of the experiment, there is considerable multicollinearity between the temperature on the one hand and the relative humidity and daylength on the other hand, making an independent assessment of these effects difficult. Nevertheless, model building showed that the main driving force in preference is temperature and not relative humidity or daylength.

Over the whole experimental period, winter morph *D. suzukii* showed a clear preference for ACV during autumn and winter (Figure 4B). This preference started shifting towards strawberries around May and the model shows a peak in preference for strawberry around August, at the end of the warmest period of the year. For summer morph *D. suzukii*, a similar evolution was observed within its limited window of occurrence. 

## 4. Discussion

In all field comparisons of trapping efficiency of synthetic lures versus ACV based baits in the present study, the typical [5,32,33,34,35,36,37] “low capture period” of ACV could be observed. In blueberry and blackberry, the weekly sample means of *D. suzukii* flies per trap with ACV remained lower than 100 until the 26th of August and the 1st of September, respectively. In the abandoned and the commercial cherry orchards, this was until the 4th of July and the 5th of August, respectively. It was noted that during this “low capture period” of ACV based baits, the synthetic lures consistently trapped significantly higher numbers of *D. suzukii*. These findings indicate that when ACV (and by extension probably other fermentation baits) traps low numbers of *D. suzukii*, this does not always reflect the fly’s true abundance [35] and does not imply that flies are just absent or inactive [32], as it is possible to trap them in larger numbers with other attractants. Although often noted as an explanation in literature [35,42,46], the presence or absence of ripe fruit appears not to be merely the reason for the “low capture period” and the “autumn peak”. We hypothesise that *D. suzukii* shows a period of fruit focused and/or fermentation cue neglecting behaviour during the whole “low capture period” which is not only mediated through spatial fruit availability. That seasonal focus might be the effect of a significant proportion of the population falling into a specific category of physiological state. 

In both laboratory and field olfactory preference experiments, we compared ACV with strawberry as a model for attractive host fruits [3,7,68,69]. ACV is created from apple juice, by a two-step fermentation process with yeasts as the first agent and acetic acid bacteria (AAB) as the second [70]. Hence, the solution has a volatile profile with compounds originating from the fruit itself, yeast fermentation and AAB fermentation. A range of symbiotic yeasts and AAB have been associated with *D. suzukii* [71,72,73,74,75,76,77,78] and many of them are known to be attractive as a protein rich food source for adults [57,72,79,80] as well as to benefit its larval development on protein-scarce fruit [78,80]. *Drosophila suzukii,* in contrast to most drosophilids, prefers fresh rather than decaying fruits for oviposition. Fresh undamaged fruits serve merely as an oviposition substrate for females [61,81] as fruit only becomes a suitable and preferred food source if the mesocarp or juice is accessible (oviposition punctures do not suffice) [37,61,82]. Since damaged or decaying fruits allow for microbial growth, it has been proposed that adult *D. suzukii* uses fermentation cues to localise food sources but that females use fresh fruit cues to find suitable oviposition substrates [69,83]. 

In our olfactometer studies, male *D. suzukii* showed a clear preference for ACV over strawberry, regardless of dietary pre-treatment or seasonal morphology. Since this is in contrast with female flies tested in the same experiments, this finding corroborates the hypothesis that fresh fruit volatiles mediate the localisation of oviposition substrates. Due to this contrast in preference between the sexes and the fact that until now no volatile pheromones of *D. suzukii* have been identified [84,85], it is assumed that the effects of simultaneously testing both sexes were limited. Further, the tested females were mated and hence might have evoked less following behaviour of the males [86]. In the feeding status experiments, starved females showed a limited preference for ACV, as a cue for food, and were markedly more responsive early in the test. Both this diverged preference and high activity of starved flies are known effects described in *Drosophila* spp. Starved flies move about more actively [87], have higher odour tracking perseverance [88] and a higher response rate when offered a single (food cue) choice [54,58] compared to fed flies. For *D. melanogaster* and *Drosophila simulans*, Turelli and Hoffmann (1988) showed in mark–release–recapture experiments that starved flies were less discriminating and prone to be attracted to inferior resources [89]. Not only could this explain the little focused preference of flies intentionally starved but also the observed levelling of preferences towards the end (24 h) of the experiment, as flies become unintentionally starved during the experiment. In this study, sugar-fed female *D. suzukii* were strongly attracted to ACV whereas artificial medium-fed females were significantly less so (and hence were more attracted to the fresh fruits). It is not surprising that sugar-fed flies were attracted to a strong nutritional (protein) cue, given that *D. melanogaster* shows compensational behaviour when deprived of essential nutrients [49,52,90]. Medium-fed flies have no incentive for nutrient balancing behaviour and therefore have a less pronounced preference for ACV. These fully fed reproductive females will have a larger focus on reproductive behaviour hence choosing more for the fruits (oviposition substrate). It is worth noting that the dietary pre-treatment resulted in a significantly higher (about 3:2 ratio) number of vitellogenic oocytes in medium-fed flies as compared with both sugar-fed and starved flies. 

In the reproductive status experiments, we attempted to generate female *D. suzukii* of the same age that only differed in the status of oogenesis, with a “24h protein repletion” treatment being able to correct for the aforementioned nutritional balancing behaviour. Protein deprived (sugar-fed) flies and flies with a temporary or permanent supply of artificial media all differed by the number of vitellogenic oocytes, but only protein deprived (sugar-fed) females displayed a strong preference for ACV. It therefore remains unclear if this preference is solely due to nutrient compensational behaviour or whether there is another effect related to the oogenic stage. Wong et al. (2018) showed that in a triple choice (raspberry essence, fermenting bait and water) laboratory experiment there was a relation between the bait preference and the number of mature eggs in the ovaries of *D. suzukii*. Flies of different reproductive status were generated like in the present study but without the “nutritional balanced” intermediate, making it less clear whether to attribute this relation to the reproductive state or nutritional balancing [58]. However, the relation between egg development and the interest for either food or fruits is supported by Swoboda-Bhattarai et al. (2017), who showed that *D. suzukii* females collected in-field on fruits had significantly more mature eggs in the ovaries than those collected in traps with fermenting baits [91]. In the present study, despite the high statistical power (n = 1235 over all experiments, excluding virgins and n = 350 for reproductive status experiments) we failed to find a relationship between the number of mature eggs in the ovaries and uterus and the preference for the fermentation versus fruit cues after 24 h. It is probable that the “slow” responsiveness in our test due to the assumed low concentration of volatiles (a limitation of using real fruits instead of concentrated extracts) and the aforementioned preference-levelling effect due to starvation during the 24h testing period resulted in the inconsistencies between our findings and the earlier studies. 

The mating status of *D. suzukii* females had a clear effect on the preference between fruit and fermentation volatiles, with mated females preferring the strawberries and virgin females the ACV. In order to eliminate effects of age or reproductive state, the virgins in these experiments were 7 days old and had the full complement of unfertilised eggs. For *D. melanogaster*, it is known that these unfertilised eggs only differ from fertilised eggs in the embryogenesis, with vitellogenesis continuing at about the same rate [92,93], hence similar nutrient requirements are expected. Due to the low number of males surviving winter [34] and the females’ specialised (large and expandable) spermathecae [32], *D. suzukii* was thought to mostly overwinter as mated females [6,32,34]. Grassi et al. (2018) recently showed that in Trentino (Northern Italy), the percentage of female *D. suzukii* with sperm stored in their spermatheca and/or seminal receptacle and the percentage of males with sperm in their testes drops in winter to about 30 and 40%, respectively [65]. This proves that, although autumn mating and sperm storage is most favourable for population restoration [93], a large percentage of females overwinters as virgins. These virgin females depend on spring mating with the few available males or the progeny of the mated females. The capacity to postpone egg-laying in the absence of males, referred to as “virgin egg retention” is genetically determined in *D. melanogaster*. Strains of the latter species with long “virgin egg retention” have the best chances of surviving winter (i.e., surviving longer than their mated counterparts) as they reduce waste material (eggs) [93,94]. Our observations suggest that *D. suzukii* has a “virgin egg retention” period of at least 7 days, which is already in the long range of existing phenotypes of *D. melanogaster* [93]. This “virgin egg retention” may be an explanation for the clear preference we observed in *D. suzukii* virgins for fermentation volatiles (food) over fruit volatiles (oviposition substrate), in sharp contrast to mated females. 

The olfactometer experiments on seasonal morphology demonstrated clear preferences of summer morph females for fruit volatiles whereas winter morphs show no particular preference. Winter morphs in these experiments were acclimated and were of the same reproductive state as the summer morphs, although a small but significant difference in vitellogenic oocyte load was noted, suggesting that quiescence effects did not play a role in this behaviour. Kirkpatrick et al. (2018) [56] described that winter morphs of *D. suzukii* (similarly laboratory generated, without mention of an acclimation period before the tests) showed a general reduced antennal response in electroantennography experiments. Moreover, in comparison to summer morphs, a significantly higher response of winter morphs to geosmin in no-choice bio-assays and a trend of reduced avoidance to geosmin in T-maze assays was reported [56]. As geosmin is a moderate repellent for *D. suzukii* [95,96] these findings indicate a level of indiscriminating olfactory behaviour of winter morph *D. suzukii* which could explain its lack of preference in our experiments. 

The outcome of the year-round field preference experiment is in line with the abovementioned olfactometer experiments with winter and summer morphs of *D. suzukii*, with summer morphs displaying a higher preference for fruit volatiles than winter morphs. The field experiment reveals, however, that seasonal morphology does not explain the seasonal change in olfactory preference, as both follow an almost parallel trend. Based on the laboratory results with males consistently preferring fermentation cues, sex was expected to also appear as an important factor influencing preference in the field. As this was, however, not the case, one could speculate that male flies might be following virgin females into the traps (which was never possible in the laboratory experiments). It deserves attention that the volatile profile of the strawberries used in the field experiment certainly underwent changes during the trapping interval. During summer the strawberries were probably increasingly emitting ethanol and esters as (m)ethyl acetate [97] and/or fungal produced volatiles as 1-octen-3-ol [98]. Nevertheless, the shift in preference over the year is unmistakable and to our best knowledge never shown before. This shift confirms the widely described hypothesis that during its typical “low capture period” with fermentation baits, the preference of *D. suzukii* flies shifts to fruits almost exclusively. The unambiguous preference of *D. suzukii* for fermentation volatiles (ACV) during autumn, winter and spring could be explained by the reproductive, nutritional, and mating status of the females and most probably a combination of all these factors. When linking our results on the field preference with the seasonal reproductive status described by Panel et al. (2018) for the same year (2017) in a neighbouring country (The Netherlands), it seems that the shift from previtellogenic *D. suzukii* females to vitellogenic females more or less coincides with the preference shift in our work [64]. A nutrient-balancing effect may be at play in spring and winter when deprivation for macro-nutrients is expected [63,99] but is probably highly correlated with the reproductive state. In autumn, rotting fruits may be ubiquitous [100] and feeding status seems less influential, but *D. suzukii* does enter reproductive quiescence. It is possible that the shift in preference from fermentation cues to fruit cues in spring is postponed by overwintering virgin females that carry mature, unfertilised eggs but have no interest in fruit cues yet (as shown in our olfactometer experiments). 

Our findings could lead to better monitoring strategies and a more efficient development of behavioural control methods like bait sprays and mass trapping. Up to now, many screenings of new candidate attractants have been done during autumn when the density of *D. suzukii* is high and flies are more easily trapped with most attractants [101,102]. In that way, attractants and compounds are selected that perform specifically well during autumnbut will probably againlead to “low capture periods” during summer. The findings of the present study could also be relevant for developing prediction models for *D. suzukii* [103].

## 5. Conclusions

In the present study we showed in different ways that *D. suzukii* populations undergo a seasonal shift in olfactory preference from fermentation cues during autumn, winter and spring to fruit cues during summer. Our olfactometer experiments suggest that the preference between fermentation and fruit volatiles is strongly determined by the feeding, mating and reproductive status of the females. Well-fed reproductive summer morph females clearly prefer host fruit odours in the olfactometer experiments. In the field preference experiment, both winter and summer morph *D. suzukii* strongly preferred fruit volatiles over fermentation volatiles during summer, when females of both morphs are reproductive. These findings indicate that during summer *D. suzukii* primarily are in search of oviposition sites. Consequently, our findings have important implications for the development of mass trapping and Attract and Kill (A&K) strategies and indicate that there might not exist one ultimate attractant for *D. suzukii* which covers the whole season. Since the olfactory preference of *D. suzukii* flies is dependent on their physiological status which in population terms varies throughout the year, attractant blends for monitoring, A&K or mass trapping strategies should consist of both fermentation cues and host fruit mimicking odours. This would allow to catch/target both the nutrient deprived founder females and the well-fed, reproductive, fruit infesting females. 

## Supplementary Materials

The following are available online at https://www.mdpi.com/2075-4450/10/7/200/s1, Figure S1: A field preference experiment was done from the 14th of October 2016 till the 22nd of December 2017, the preference for strawberries (over ACV) in relation with the mean relative humidity during the trapping interval is depicted. Dots are the estimated values from the model, triangles the actual observations. The line is a local regression line (LOESS) and does not represent the underlying logistic function, Figure S2: A field preference experiment was done from the 14th of October 2016 till the 22nd of December 2017, the preference for strawberries (over ACV) in relation with the mean daylength during the trapping interval is depicted. Dots are the estimated values from the model, triangles the actual observations. The line is a local regression line (LOESS) and does not represent the underlying logistic function, Table S1: Mean responsiveness (%) ± SD of *D. suzukii* in olfactometer experiments for each treatment/status and each assessment time (3, 6 and 24 h).
