# Supplementary material for: Olfactory Preference of Drosophila suzukii Shifts between Fruit and Fermentation Cues over the Season: Effects of Physiological Status
Source: Insects. 2019 Jul 6;10(7):200. doi: 10.3390/insects10070200 (PMC6681279; doi:10.3390/insects10070200)
Supplement: Supplementary file 1 [file insects-10-00200-s001.pdf]

insects

Article

Olfactory Preference of *Drosophila suzukii* Shifts between Fruit and Fermentation Cues over the Season: Effects of Physiological Status

Rik Clymans, Vincent Van Kerckvoorde, Eva Bangels, Wannes Akkermans, Ammar Alhmedi, Patrick De Clercq, Tim Beliën and Dany Bylemans

Supplementary Materials

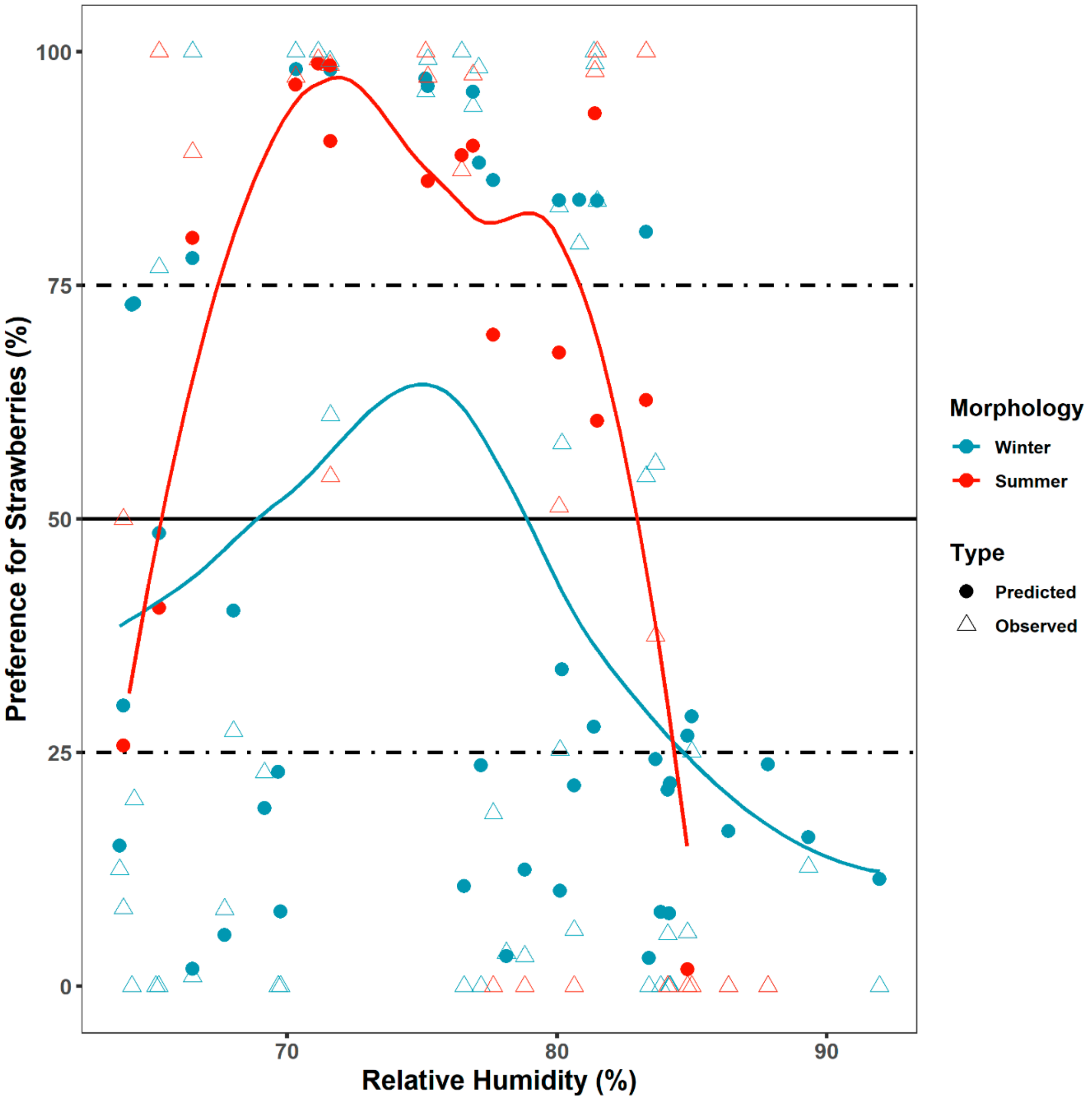

Figure S1. A field preference experiment was done from the 14th of October 2016 till the 22nd of December 2017, the preference for strawberries (over ACV) in relation with the mean relative humidity during the trapping interval is depicted. Dots are the estimated values from the model, triangles the actual observations. The line is a local regression line (LOESS) and does not represent the underlying logistic function.

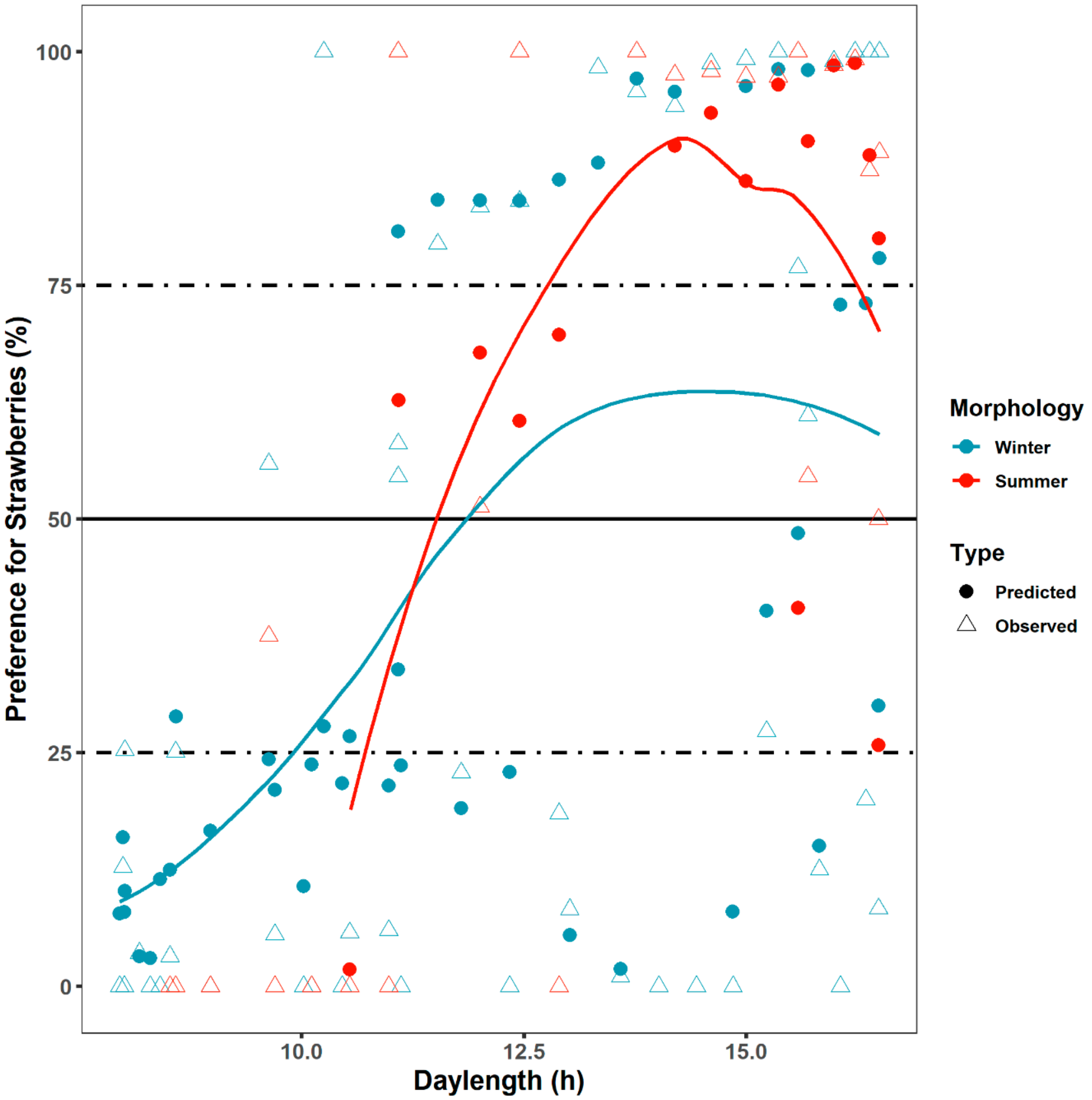

Figure S2. A field preference experiment was done from the 14th of October 2016 till the 22nd of December 2017, the preference for strawberries (over ACV) in relation with the mean daylength during the trapping interval is depicted. Dots are the estimated values from the model, triangles the actual observations. The line is a local regression line (LOESS) and does not represent the underlying logistic function.

Table S1. Mean responsiveness (%) ± SD of *D. suzukii* in olfactometer experiments for each treatment/status and each assessment time (3, 6 and 24 hours).

Experiment cluster	Treatment/ Status	Responsiveness (%)						n
		Females			Males			
		3 h	6 h	24 h	3 h	6 h	24 h	
Feeding status	Medium	9.2 ± 9.0	10.8 ± 10.0	87.7 ± 13.1	2.4 ± 4.4	4.1 ± 5.1	81.3 ± 12.3	12
	Sugar	7.9 ± 9.3	9.8 ± 10.9	85.6 ± 10.1	1.9 ± 4.4	4.6 ± 7.8	78.8 ± 14.3	12
	Water	29.8 ± 20.0	49.9 ± 22.2	89.7 ± 13.3	35.0 ± 23.5	48.1 ± 28.1	74.1 ± 22.4	12
Seasonal morphology	Summer morph	28. 8± 19.9	43.8 ± 20.3	93.9 ± 7.8	10.5 ± 9.0	25.3 ± 17.0	91.2 ± 11.2	18
	Winter morph	18.2 ± 14.1	31.0 ± 18.0	89.7 ± 14.3	13.7 ± 11.1	25.8 ± 14.8	92.2 ± 12.3	18

Mating status	Mated	7.8 ± 8.1	14.6 ± 13.3	94.4 ± 9.2	18
	Virgin	3.9 ± 7.8	13.0 ± 14.0	96.5 ± 5.1	18
Reproductive status	Protein fed	10.4 ± 10.2	26.0 ± 21.4	96.7 ± 7.8	12
	Protein deprived	10.8 ± 9.0	28.9 ± 14.8	89.4 ± 12.0	12
	24h protein repletion	13.1 ± 12.6	27.7 ± 15.2	91.7 ± 10.3	12

© 2019 by the authors. Submitted for possible open access publication under the terms and conditions of the Creative Commons Attribution (CC BY) license (http://creativecommons.org/licenses/by/4.0/).
